# High-Precision Lensless Microscope on a Chip Based on In-Line Holographic Imaging

**DOI:** 10.3390/s21030720

**Published:** 2021-01-21

**Authors:** Xiwei Huang, Yangbo Li, Xuefeng Xu, Renjie Wang, Jiangfan Yao, Wentao Han, Maoyu Wei, Jin Chen, Weipeng Xuan, Lingling Sun

**Affiliations:** Ministry of Education Key Lab of RF Circuits and Systems, Hangzhou Dianzi University, Hangzhou 310018, China; 181040007@hdu.edu.cn (Y.L.); xuxuefeng@hdu.edu.cn (X.X.); wangrenjie@hdu.edu.cn (R.W.); yaojiangfan@hdu.edu.cn (J.Y.); wentaohan@hdu.edu.cn (W.H.); lucienwell@hdu.edu.cn (M.W.); chenjin272@hdu.edu.cn (J.C.); xuanweipeng@hdu.edu.cn (W.X.); sunll@hdu.edu.cn (L.S.)

**Keywords:** high-resolution, on-chip imaging, lensless holographic microscope, phase recovery, POCT

## Abstract

The lensless on-chip microscope is an emerging technology in the recent decade that can realize the imaging and analysis of biological samples with a wide field-of-view without huge optical devices and any lenses. Because of its small size, low cost, and being easy to hold and operate, it can be used as an alternative tool for large microscopes in resource-poor or remote areas, which is of great significance for the diagnosis, treatment, and prevention of diseases. To improve the low-resolution characteristics of the existing lensless shadow imaging systems and to meet the high-resolution needs of point-of-care testing, here, we propose a high-precision on-chip microscope based on in-line holographic technology. We demonstrated the ability of the iterative phase recovery algorithm to recover sample information and evaluated it with image quality evaluation algorithms with or without reference. The results showed that the resolution of the holographic image after iterative phase recovery is 1.41 times that of traditional shadow imaging. Moreover, we used machine learning tools to identify and count the mixed samples of mouse ascites tumor cells and micro-particles that were iterative phase recovered. The results showed that the on-chip cell counter had high-precision counting characteristics as compared with manual counting of the microscope reference image. Therefore, the proposed high-precision lensless microscope on a chip based on in-line holographic imaging provides one promising solution for future point-of-care testing (POCT).

## 1. Introduction

Optical microscopes play a significant role in scientific study and clinical testing in modern medicine and life sciences, as they can expand and identify morphological features of samples (including cells, bacteria, marine microorganisms, etc.) that cannot be enlarged and seen by the naked eyes. With the iterative upgrade of the spatial resolution of optical microscopes, microscopes with various extended functions, including scattering, absorption, refraction, phase modulation, etc., have also emerged so that the morphological characteristics of transparent samples [1,2,3,4,5,6], the subtle internal changes of thick samples [7,8,9], and structural details even smaller than the wavelength of the light source [10,11] can be easily obtained. However, due to large size, high cost, and complicated operation, these microscope detection platforms can only be used in advanced laboratories and medical institutions. Hence, their scope and conditions of application are limited, especially in resource-limited areas where infectious diseases such as tuberculosis, malaria, sickle cell virus infection, and other diseases are common. Therefore, the use of portable optical microscopes in resource-limited areas to improve detection efficiency is vital for preventing the spread of infectious diseases [12,13,14].

To meet such needs, in recent years, the combination of Complementary Metal Oxide Semiconductor (CMOS) image sensor and microfluidic technology provides an opportunity for the development of portable on-chip imaging detection systems, and significant achievements have been made in the field of lensless microscope research in the recent decade [15,16,17,18,19,20]. The reasons are as follows: First, with the advancement of semiconductor processing technology and the replacement of Charge-coupled Device (CCD) image sensors by CMOS image sensors as image capturing elements, the diameter of the pixel pitch is getting smaller and smaller, and the pixel density on the silicon substrate of 20~30 mm^2^ has become higher. The impact of lower pixel pitch (for example, Sony’s IMX586 color pixel diameter is 0.8 µm) greatly improves the image resolution. Second, lensless microscopes rely heavily on numerical calculations to recover high-resolution images. With the advancement of semiconductor technology, the computing power of processing chips (such as Central Processing Unit (CPU), Graphics Processing Unit (GPU), Digital Signal Processing (DSP), Field Programmable Gate Array (FPGA), etc.) continues to increase, while prices and power consumption continue to decline, and this is conducive to low-cost, portable imaging systems. Third, algorithms to improve image resolution, such as compression and decompression algorithms [21], image synthesis algorithms [22,23], as well as machine learning algorithms to identify samples quickly [24,25], have been successfully applied in lensless microscope systems. According to imaging principles, lensless imaging systems can be divided into shadow imaging, fluorescence imaging, holographic imaging, etc.

The lensless shadow imaging system, which directly uses the shadow pattern of the sample illuminated by the light source for analysis, is the most basic imaging method. For example, our previous work proposed a lensless microfluidic miniaturized flow cytometer with super-resolution processing based on shadow imaging, which uses online machine learning to perform super-resolution reconstruction of a single frame, and compared with the cytometer, the particle counting error is less than 8% [26]. Zheng et al. proposed a sub-pixel perspective scanning microscope (SPSM), which can obtain two shadow images with a small distance by changing the position of the light source, and then high-resolution images are synthesized by sub-pixel shifted low-resolution pictures at a high rate, achieving a resolution of 660 nm [27]. However, the resolution of the shadow imaging method is still limited, and complex algorithms are required for super-resolution reconstruction in the later stages.

The lensless fluorescence imaging system uses light waves of appropriate wavelengths to irradiate dyed fluorescent samples and evaluates the proportion of specimens by comparing different color reactions on the screen. It can be used in white blood cell classification and the detection of rare cells in the blood, such as circulating tumor cells. For example, Coskun and colleagues proposed a large field-of-view (FOV), dual-imaging mode lensless fluorescent on-chip imaging system, which can achieve high-throughput detection and detection of rare cells in a large FOV [28]. Yet, the fluorescence imaging method requires the addition of filters and other parts to the experimental device, which increases the complexity and system cost.

A lensless holographic imaging system uses a coherent or incoherent light source to obtain a ring pattern formed by the interference of the reference light wave and the object wave. Since holographic imaging contains more information about the object, the characteristics of the sample can be reconstructed by the principle of Fresnel integral diffraction, which provides excellent help for sample analysis. Seo et al. used a lensless holographic imaging platform to implement an on-chip cell counter and used a custom pattern recognition algorithm to characterize and count the cells of interest in the sample. Their experiments verified that the lensless holographic imaging not only improved the signal-to-noise ratio but also had a good imaging ability for weakly scattered samples [29]. Vercruysse et al. proposed a lensless online holographic microscope to realize the differential recognition of unlabeled white blood cell subtypes (lymphocytes, monocytes, granulocytes), which was consistent with the results obtained by conventional blood analyzers [30]. Wu et al. used the Generative Adversarial Networks (GAN) network to reconstruct the sample patterns at different levels in the three-dimensional (3D) sample from a single hologram, and the results matched the slice performance of the high Numerical Aperture (NA) bright-field microscope [31]. Fang et al. have developed an on-chip lensless flow cytometer, which uses the holographic imaging principle and the broad FOV of the S-shaped pipe to achieve a cell counting error of less than 2% [32]. Since the experimental device of holographic imaging is simple, and the resolution can be improved through phase recovery, it has been widely studied and applied in recent years [33,34,35].

Therefore, to overcome the shortcomings of shadow imaging and fluorescence imaging, this paper demonstrates an alternative method of lensless on-chip holographic imaging towards telemedicine applications. It is a portable high-precision detection device based on lensless holographic imaging to capture cells on the glass smear, and uses machine learning to classify and count mouse ascites tumor cells and polystyrene particles in holographic phase recovery images. Experiments show that the resolution of the platform is 1.41 times that of the shadow imaging platform, and the accuracy of cell counting is similar to the manual counting results of the microscope reference image. Besides, the platform has no complex optical path system, simple operation, small size, no damage to samples, low cost, and can be applied in resource-poor areas for point-of-care testing (POCT), which has specific application prospects in preventing the spread of diseases and early detection.

## 2. Methods

### 2.1. System Setup

**Figure 1:**
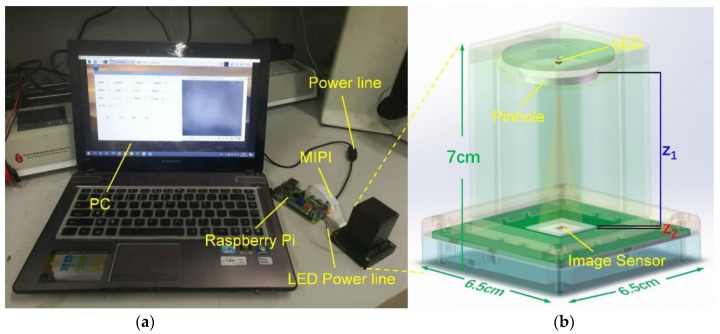
Schematic of the lensless on-chip microscope. (**a**) Photographs of the apparatus, (**b**) structure of in-line holographic imaging device.

This system is built using the imaging principle of in-line holography, and the overall structure is shown in Figure 1. The system mainly consists of a CMOS image sensor (IMX219PQ, Sony, Tokyo, Japan, 1.12 μm pixel size, 3280 H × 2464 V), a yellow Light Emitting Diode (LED) light source (LY-E65B, OSRAM Opto Semiconductors Inc., Regensburg, Germany, λ = 587 nm), and a pinhole (100 μm in diameter). To make full use of the imaging area of the image sensor, a cover glass (with a thickness of 0.5 mm) is directly covered on the image sensor and glued with liquid glue to prevent misalignment due to moving the experimental device. It not only achieves the conditions of static imaging but also controls the distance between the cover glass and the image sensor in a small range so that the magnification ratio of the sample imaging pattern can also be controlled to avoid imaging distortion. In order to obtain a holographic interference pattern on the surface of the image sensor, the LED light is controlled at a distance of 5 cm from above the image sensor. Besides, the pinhole is placed under the LED to improve the coherence of the LED light source. All the experimental structural components are finally assembled by a 3D-printed dark box (6.5 × 6.5 × 7 cm^3^) to form a miniaturized on-chip lensless holographic imaging detection system. Note that the LED is powered by an external Raspberry Pi circuit board through a power line. The Raspberry Pi board is also connected with the CMOS image sensor board through an Mobile Industry Processor Interface (MIPI) interface. The captured holographic images are then transferred to the PC via the Raspberry Pi for further analysis. 

### 2.2. Sample Preparation

In order to evaluate the imaging effect of the holographic imaging platform, we prepared two different experimental samples. One is the optical positive film resolution plate (USAF-1951, Yaopu Optics, Chengdu, China), which is used to measure the resolution of the imaging system. There are some particular patterns on the resolution board, and its line width and interval have been precisely set. By identifying the most indistinguishable reference line, the resolution capability of the system can be determined. The resolution board was cleaned by wipe and placed above the CMOS image sensor for testing. Another is the mixed solution of mouse ascites tumor cells and polystyrene particles (15 μm). First, some suspended malignant tumor cells were inoculated into the abdominal cavity of mice. A few days later, the cells proliferated in the abdominal cavity of the mice to produce a large number of offspring to form mice with ascites tumors, and ascites were drawn for later use. In order to ensure that clear wavefront information was observed in the coaxial holographic experiment, we diluted the mixed solution, extracted 1 μL of mouse ascites and 1 μL of polystyrene particles to mix, and then extracted 200 μL of phosphate buffered solution (PBS) and mixed with 2 μL of the cell solution. The diluted solution was made into a glass smear for testing.

### 2.3. Imaging Principle

In the on-chip lensless holographic imaging detection system, a translucent sample is placed above the image sensor, usually $z_{2}$ (<1 mm) from the image sensor. An incoherent light source is fixed at a distance $z_{1}$ from the sample, and a pinhole is set directly below the light source so that the sample is irradiated by the light source and instantly forms a holographic pattern on the image sensor, and the image sensor encodes the holographic pattern by intensity.

The on-chip lensless holography is derived from the holography invented by Gabor in 1948 [36]. This method not only records the amplitude information of the light field but also records the phase information. Therefore, holography records the complete information of an object. In the holographic recording process, the recording medium records the complex amplitude, that is, simultaneously records the amplitude and phase information of the original object light wave. The intensity change recorded in this way is called a hologram.

In in-line holography, assuming that the object t is semi-transparent, it can be approximated as:

(1) $$t\left(x_{0},y_{0}\right)=1+\Delta t\left(x_{0},y_{0}\right)$$

Here, Δt is the transmittance fluctuation and Δt<<1. When an object is locally illuminated by plane wave A, it will coherently propagate $z_{2}$ distance (distance from the sample to the image sensor):

(2) $$P\left[z_{2}\right]\left\{A\cdot t\left(x_{0},y_{0}\right)\right\}=P\left[z_{2}\right]\left\{A\right\}+P\left[z_{2}\right]\left\{A\cdot \Delta t\left(x_{0},y_{0}\right)\right\}=A^{\prime }+a\left(x,y\right)$$

Here, $P\left[z_{2}\right]\left\{*\right\}$ is the transfer function acting on $z_{2}$, ε and η are frequency domain coordinates, and λ is the wavelength of the light source.

(3) $$P_{z_{2}}\left(\varepsilon ,\eta \right)=\{\begin{array}{c} \exp\left[j\cdot z_{2}\cdot \frac{2\pi }{\lambda }\cdot \sqrt{1-{\left(\lambda \varepsilon \right)}^{2}-{\left(\lambda \eta \right)}^{2}}\right],\;\varepsilon^{2}+\eta^{2}<\frac{1}{\lambda^{2}}\\ 0 \end{array}$$

On the image sensor plane, the object light wave a(x,y) passing through the object interferes with the reference light wave $A^{\prime }$ to form a hologram, and the intensity information I(x,y) is recorded:

(4) $$I\left(x,y\right)={\left|A^{\prime }+a\left(x,y\right)\right|}^{2}={\left|A^{\prime }\right|}^{2}+A^{\prime *}\cdot a\left(x,y\right)+A^{\prime }\cdot a^{*}\left(x,y\right)+{\left|a\left(x,y\right)\right|}^{2}$$

In (4), the second and third items are related to the reconstruction of object information. The first item is the reference light information, which can be removed by a sample-free background image, the fourth item is the object self-interference information, it can be ignored when ∆t<<1, because for holography, it does not include any useful information, and during the holographic reconstruction process, the focused image of the object and the out-of-focus image will overlap, and the twin image will form. In the on-chip holographic imaging, $z_{2}\;$is usually very small. The twin image has a severe impact on the image of the actual object, so it needs to be removed by related methods.

For digital holographic imaging, according to the angular spectrum theory, sample information, including amplitude and phase, can be decoded by back propagation:

(5) $$P\left[-z_{2}\right]\left\{A^{*}\cdot I\left(x,y\right)\right\}={\left|A\right|}^{2}\cdot \left[1+\Delta t\left(x,y\right)+P\left[-2z_{2}\right]\left\{\Delta t^{*}\left(x,y\right)\right\}\right]+P\left[-z_{2}\right]\left\{{\left|a\left(x,y\right)\right|}^{2}\right\}$$

In (5), $A^{*}$ is the conjugate of the plane wave and I(x,y) is the recorded hologram. On the right side of the Equation (5), the first term is the background light, the second term is the actual sample, the third term is the twin image interference, and the fourth term is the self-interference term backpropagation interference. For smaller or more scattered samples, the twin image interference will not cause interference to the reconstructed image, and routine analysis can be satisfied by simple backpropagation. For other samples, the existence of the twin image will interfere with the analysis of the results, making the recovered sample information unclear. Therefore, proper methods are needed to reduce the influence of the twin image, such as the phase iterative recovery method.

Regarding the recorded hologram as just ordinary diffraction field intensity information, the phase information is lost. Mudanyali et al. proposed a phase iteration method, which uses a threshold definition method to determine the boundary of the sample object [37]. After backpropagation, the twin image interference information which is outside the edge of the object can be removed iteratively by imposing constraints. The method consists of the following steps:

1. The root mean square of the holographic image information I(x,y) recorded by the image sensor is propagated back to the object surface, the phase information on the object surface is assumed to be zero at this time, and the phase information is finally restored on the object surface through an iterative method. At the same time, the threshold segmentation method is used to determine the boundary of the sample object.
  (6) $$U_{-z_{2}}^{1}\left(x,y\right)=P_{z_{2}}^{-}\left[\sqrt{I\left(x,y\right)}\right]$$
  In Equation (6), $U_{-z_{2}}^{1}$ represents the real image field distribution after the first iteration.
2. The information inside the boundary of the object is retained, and the information outside the boundary is replaced by the information after the backpropagation of the sample-free background image:
  (7) $$U_{-z_{2}}^{i+1}\left(x,y\right)=\{\begin{array}{c} m\cdot D_{-z_{2}}\left(x,y\right),\;x,y\notin S\\ U_{-z_{2}}^{i}\left(x,y\right),\;x,y\in S \end{array}$$
  In Equation (7), $D_{-z_{2}}\left(x,y\right)\;$is obtained by backpropagating the root mean square of the background image without samples, m is the iteration coefficient, and $U_{-z_{2}}^{i}$ represents the real image field distribution after the *i*th iteration.
  (8) $$m=mean\left(\frac{U_{-z_{2}}^{i}\left(x,y\right)}{mean\left(D_{-z_{2}}\left(x,y\right)\right)}\right)$$
3. The reconstructed field after being constrained in the second step is propagated forward to the surface of the image sensor. At this time, the phase value is no longer zero, and the phase value is retained. The amplitude value is determined by the root mean square band of the original recorded holographic image, $U_{0}^{0}\left(x,y\right),$ amplitude. The diffraction field, $U_{0}^{i}\left(x,y\right)$, after the *i*th iteration is expressed as follows:
  (9) $$U_{0}^{i}\left(x,y\right)=\left|U_{0}^{0}\left(x,y\right)\right|\cdot \exp\left(j\cdot \phi_{0}^{i}\left(x,y\right)\right)$$
  where $\phi_{0}^{i}\left(x,y\right)$ denotes the phase of the field after the *i*th iteration.

**Figure 2:**
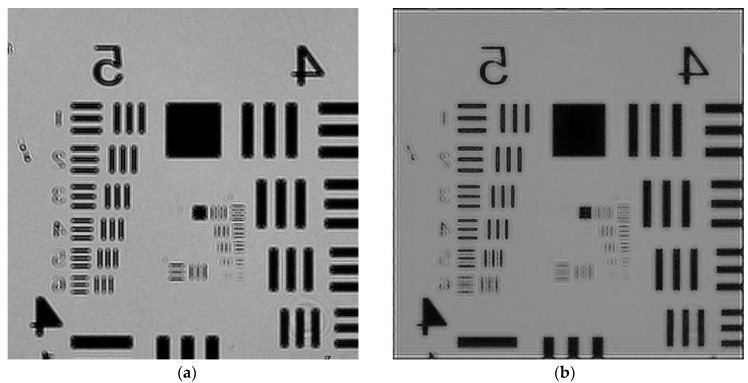
The results of iterative phase recovery. (**a**) Original United States Air Force (USAF) holographic image, (**b**) iteratively recovered image.

Steps 1 to 3 can be iterated until the phase recovery converges. Typically, the results are obtained with less than 15 iterations, and the results are shown in Figure 2b.

## 3. Experiments and Results

### 3.1. Image Quality Assessment

**Figure 3:**
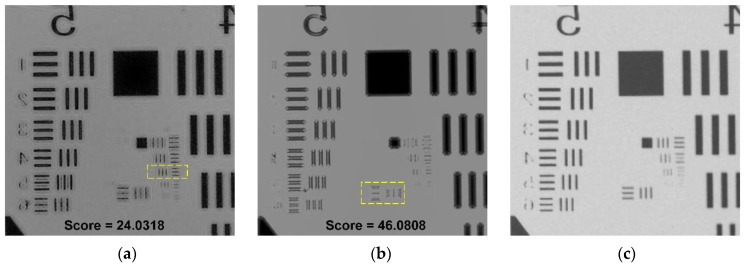
Comparison of images taken by different systems. (**a**) Iteratively recovered image, (**b**) shadow image, (**c**) microscope image.

In order to test the performance of the algorithm, we used two imaging methods, shadow imaging and holographic imaging, to shoot experiments on the United States Air Force (USAF) resolution plate. The experimental conditions were set with the same parameters. The center wavelength of the light source is 587 nm, the distance between the USAF resolution plate and the image sensor is 0.5 mm, and the pixel size is 1.12 μm. In addition, we also used a 10× objective microscope to photograph the same area of the USAF resolution plate as a comparison image. The comparison of the holographic image after iterative phase recovered, shadow image, and 10× ocular lens is shown in Figure 3.

In order to more intuitively evaluate the image quality of different imaging methods, we introduced image quality evaluation algorithms, which are divided into non-reference image quality evaluation algorithm [38] and reference-quality evaluation algorithm [39].

- Non-reference image quality evaluation

In the non-reference image quality evaluation, we used the Brisque algorithm to perform global scoring and evaluation on the holographic restored image and the shadow image. The overall principle of the algorithm is to extract the MSCN (Mean Subtracted Contrast Normalized) coefficients from the normalized image and fit the MSCN coefficients to an asymmetric generalized Gaussian distribution, extract the fitted Gaussian distribution feature, then input it into the support vector machine (SVM) for regression calculation, thereby obtaining the image quality evaluation result, and the score results are shown in Figure 3a,b. Based on the Brisque algorithm, lower score indicates better quality.

- Reference image quality evaluation

In the reference image quality evaluation, we used two evaluation parameters, namely the blur coefficient ($K_{blur}$) and quality index (Q). The 10× microscope image is used as the reference image, and the holographic recovered image and shadow image are used as the images to be evaluated. Among them, the blur coefficient is defined as follows:

(10) $$K_{blur}=\frac{S_{i,out}}{S_{i,in}}$$

Among them, the energy characteristic of the oblique edge, $S_{i}$, is

(11) $$S_{i}=\underset{i}{\sum }\underset{j}{\sum }\left|y_{f}^{i}\left(i,j,k\right)\right|$$

In (11), $y_{f}^{i}\left(i,j,k\right)\;$is the value obtained after the brightness value of the *k*th frame, the *i*th row, and the *j*th column, which are processed by an oblique space differential filter.

It can be seen that $K_{blur}$ is the ratio of the output edge energy to the input edge energy. Assuming that the image sequence has no other distortion, the range of value $K_{blur}$ is generally between 0 and 1. The closer the $K_{blur}$ value is to 1, the higher the image clarity. The result of $K_{blur}$ is shown in Table 1. The $K_{blur}$ value 0.9738 of the recovered hologram is between 0 and 1 and is closer to 1, so the quality is better.

The quality index is defined as follows:

(12) $$Q=\frac{4\sigma_{xy}\bar{x}\bar{y}}{\left(\sigma_{x}^{2}+\sigma_{y}^{2}\right)\left[{\left(\bar{x}\right)}^{2}+{\left(\bar{y}\right)}^{2}\right]}$$

In (12), where x and y are the reference image and the image to be tested respectively, $\bar{x}$ and $\bar{y}$ are pixel mean value, $\sigma_{x}^{2}$ and $\sigma_{y}^{2}$ are pixel variance, and $\sigma_{xy}$ is the standard deviation. The value range of the quality index Q is usually between [−1, 1], the closer to 1, the higher the image quality. The result of Q is shown in Table 1. The Q value of the shadow image and the recovered hologram image are both between −1 and 1, but the Q value of the recovered hologram image 0.2174 is closer to 1, so the quality of the holographic restored image is higher.

### 3.2. Cell Analysis

In the miniaturized on-chip biological detection equipment, the cell solution was dropped on a cover glass with a thickness of 0.5 mm through a pipette, and a glass smear was made and placed above the image sensor. Under the irradiation of an incoherent LED light source, the holographic diffraction pattern of the cells was recorded by the image sensor. In order to reduce the volume and cost of the equipment, we used ordinary yellow LED as the light source, and used 3D printing technology to build a dark box with a volume of 6.5 × 6.5 × 7 cm^3^. The light source was fixed to the pinhole, and the image sensor was placed at the bottom of the box. The smear was attached to the image sensor by liquid glue. After iteratively recovering the captured cell holographic image using the above image recovery algorithm, the Fiji tool is introduced to classify, identify, and count the cells.

Classification and recognition are performed by taking an image containing different cells and use the Trainable Weka Segmentation tool to mark the different cells in color [40]. Trainable Weka Segmentation is a plug-in of Fiji that combines a set of machine learning algorithms and a set of selected image features to generate pixel-based segmentation. Weka includes a set of visualization tools and algorithms for data analysis and predictive modeling and a graphical user interface (GUI) for easy access to this feature. Next, the cell images are binarized, the adhered cells are segmented using the watershed segmentation method, and then the Cell Counter plug-in is used to count the cells of the entire image and display the counting results.

### 3.3. Discussion

**Figure 4:**
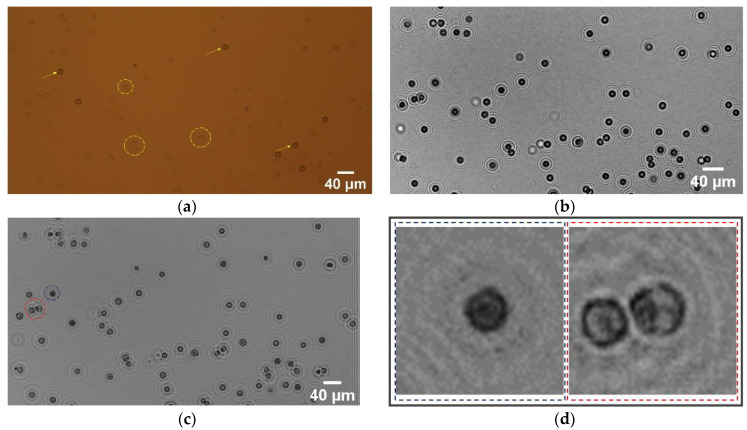
Results of the holographic imaging experiment process. (**a**) Microscope image, (**b**) raw holographic image, (**c**) iteratively recovered image, (**d**) insets of the iteratively recovered image.

In order to test the imaging performance of the designed holographic imaging system, we captured the holographic images of mixed solution of mouse ascites tumor cells and polystyrene particles (15 μm). The same chosen area was shown for comparison. Figure 4a is a microscopic image under 10× objective. In this figure, the arrows point to the polystyrene particles, and the dotted circles indicate the cells. The holographic imaging experiment process was as follows: First, a background image was taken without any sample solution to use the phase recovery algorithm to remove the background, second, a pipette was used to drop 10 μL of the mixed solution onto a cover glass with a thickness of 0.5 mm and generate the cell smears, from which the holographic imaging device would capture the images of mixed sample solution (Figure 4b). Third, the self-designed holographic phase recovery algorithm based on Matlab was employed to reconstruct the captured holographic image (Figure 4c). The results show that in the cell image after holographic phase recovery, the gray value of the low refractive index cells is generally higher than that of the high refractive index polystyrene particles, and the contour and sharpness of the cell edges is better recovered. The inset in Figure 4d, enclosed with the dashed rectangle, shows the polystyrene particle and cells after iterative phase recovery algorithm, further validating the imaging performance of our holographic microscope.

**Figure 5:**
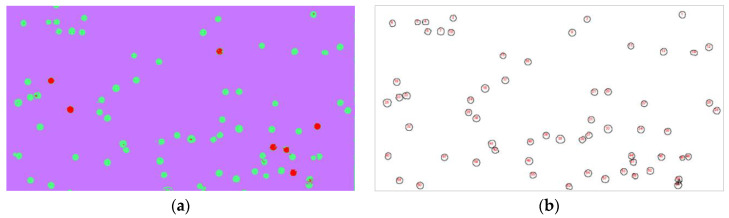
Results of the image process: (**a**) recognition results, and (**b**) counting results of the cell.

The image processing flow consists of two steps: The first step is cell identification and segmentation. We used Fiji’s Trainable Weka Segmentation plug-in to train the classifier. A small number of cells, particles, and backgrounds in Figure 4c are first selected and set into three different labels. Each label generally contains sampling information of 3–4 different positions. The classification results can be obtained through training, as shown in Figure 5a. Among them, the red parts represent polystyrene particles, the green parts represent mouse ascites tumor cells, and the purple parts represent the background. The second step is the cell counting. First, the particles and cells with incomplete edges in Figure 5a were removed by setting a threshold, then we binarized the image and filled in the holes, then broke the adherent cells, and finally counted the remaining cells in the image. The counting result is shown in Figure 5b. It can be seen from the results that the number of cells is 62, and the count value is marked inside the cell edge. The count result and manual count result of reference are similar to each other, which preliminarily verifies the accuracy of the on-chip holographic microscope.

## 4. Conclusions

In this paper, we demonstrated a lensless on-chip holographic imaging platform using in-line holography technology and used an iterative phase recovery algorithm to achieve the phase recovery of the holographic image. The resolution of the reconstructed hologram image was improved by 1.41 times compared with the traditional shadow imaging method, and the image sharpness was also improved. In the reconstructed hologram (Figure 3a), all the bars of group 6, element 4, can be identified (90.5 Line Pairs/mm), but in the traditional shadow image (Figure 3b), only group 6, element 1, can be completely identified (64 Line Pairs/mm), and the edge is out of focus. Moreover, compared with the traditional manual counting of blood smears, this paper realizes the function of automatically identifying and counting cells. Finally, because the system is small in size and low in price, it can be promoted and used in poor and remote areas and has the application prospect of point-of-care testing (POCT).

## Figures and Tables

**Table 1:** The results of reference image quality evaluation.

Image	$K_{blur}$	Q
Shadow	2.1841	0.1648
Recovered hologram	0.9738	0.2174

## Data Availability

Data available on request due to privacy restrictions.
